# Organ culture of seminiferous tubules using a modified soft agar culture system

**DOI:** 10.1186/s13287-018-0997-8

**Published:** 2018-09-26

**Authors:** Keykavos Gholami, Gholamreza Pourmand, Morteza Koruji, Sepideh Ashouri, Mehdi Abbasi

**Affiliations:** 1 Department of Anatomy, School of Medicine, Tehran University of Medical Sciences, Tehran, Iran; 2 Urology Research Center, Sina Hospital, Tehran University of Medical Sciences, Tehran, Iran; 3 Cellular and Molecular Research Center & Department of Anatomical Sciences, Iran University of Medical Sciences, Tehran, Iran

**Keywords:** Seminiferous tubules, Testicular cells, Soft agar culture system, Differentiation, Proliferation

## Abstract

**Background:**

In-vitro spermatogenesis in mammalian species is considered an important topic in reproductive biology. New strategies for achieving a complete version of spermatogenesis ex vivo have been conducted using an organ culture method or culture of testicular cells in a three-dimensional soft agar culture system (SACS). The aim of this study was to develop a new method that supports spermatogenesis to the meiotic phase and morphologically mature spermatozoa through the culture of testicular cells and seminiferous tubules (STs) in a modified SACS, respectively.

**Methods:**

First, enzymatically dissociated testicular cells and mechanically dissociated STs of neonatal mice were separately embedded in agarose and then placed on the flat surface of agarose gel half-soaked in the medium to continue culture with a gas-liquid interphase method.

**Results:**

Following 40 days of culture, the meiotic (*Scp3*) and post-meiotic (*Acr*) gene expression in aggregates and STs was confirmed by real-time polymerase chain reaction. These results were complemented by immunohistochemistry. The presence of morphologically mature spermatozoa in the frozen sections of STs was demonstrated with hematoxylin and eosin staining. We observed *Plzf*- or *Integrin α6*-positive spermatogonia in both cultures after 40 days, indicating the potency of the culture system for both self-renewal and differentiation.

**Conclusions:**

This technique can be used as a valuable approach for performing research on spermatogenesis and translating it into the human clinical setting.

## Background

Spermatogenesis is a complex process that is regulated by the endocrine system and, locally, by multiple interactions between developing germ cells and the surrounding environment through autocrine/paracrine factors [1–3]. For the last few decades, there has been much effort to develop a two-dimensional (2D)-based culture system capable of supporting all stages of spermatogenesis. Although a huge amount of information on the mechanisms and the factors that influence the process of spermatogenesis, such as growth factors, hormones, and temperature, has been gleaned from 2D/conventional culture systems [4–6], it is now evident that highly specialized microenvironments which are essential prerequisites for setting up in-vitro spermatogenesis methods cannot be reconstructed by conventional culture systems [2]. Nowadays, three-dimensional (3D) culture systems and organ culture have been developed by researchers to model testicular microenvironments ex vivo [7–9]. Recently, the importance of 3D culture systems in providing a suitable support for interaction and aggregation of germ cells and Sertoli cells has been documented by several publications. Single-cell suspension from testicular tissues embedded in soft agar [9], nanofiber scaffolds [10, 11], collagen [12], and alginate [13, 14] has been induced to trigger both cell proliferation and differentiation pathways. Although 3D culture systems provide a better understanding of the effect of cellular interactions on the process of spermatogenesis, establishment of a local gradient of paracrine/autocrine factors in 3D culture systems has not been considered, playing an essential role in the compartmentalization within the seminiferous tubules [2, 15].

Maintaining the spatial arrangement and microenvironment composition of germ cells, organ culture has also been used to generate fertile sperm [8]. Despite the success of this method in the production of functional sperm, disruption in tissue perfusion is one of the disadvantages of organ culture which results in loss of spermatogonial stem cells (SSCs) caused by hypoxia [16, 17]. Ischemic injury that occurs during culture is an important factor for the outcome of tissue culture [18]. Due to the role of ischemic stress in tissue apoptosis or necrosis, different approaches have been investigated to minimize the side effects of ischemia, such as adding antioxidants and growth factors to the medium or using high concentrations of oxygen [19, 20]. To maintain spatial arrangements and prevent hypoxia within tissues, previous studies have documented that cellular clusters or very small quantities of mechanically disintegrated tissue fragments have greater spermatogenic potential [21–23]. It thus seems that the greatest spermatogenic success takes places in cultures of small tubule fragments without disruption in the diffusion of nutrients and gases, and also cell interaction. Since diffusion of gas and nutrients is completely dependent on the thickness of the tissue and surface available for diffusion [24–26], using seminiferous tubules (STs) with more surface area and less thickness (150 μm in mice) rather than tissue fragments (size 1–3 mm^3^) can be more effective in reducing the oxidative stress caused by hypoxia. In the present study, and with the aim of reducing hypoxic effects in tissue culture and improving culture medium flow in a soft agar culture system (SACS), we attempted to develop a new culture system compatible with both seminiferous tubule culture and one suitable for aggregation of enzymatically dissociated testicular cells in the form of a 3D structure. We defined two conditions of culture: STs or testicular cells embedded in agarose separately. Treatments were placed on the flat surface of agarose gel half-soaked in the medium to use the culture with a gas-liquid interphase method.

## Methods

### Testicular cell isolation

Animal experiments in this study were approved by the ethics committee of Tehran University of Medical Sciences in accordance with the university’s guidelines. Testicular cells were obtained from 2- to 6-day-old NMRI male mice. Testes were removed from the scrotum and the tunica albuginea of the testis was removed to obtain the testis tissue. The testis tissues were cut into small pieces and transferred to the digestion solution containing collagenase type IV (1 mg/ml; Sigma, Germany), DNase (10 μg/ml; Sigma, Germany), and hyaluronidase (0.5 mg/ml; Sigma, Germany) for 20 min at 37 °C in a 5% CO_2_ incubator. The cells were dispersed by pipetting every 2–5 min until the tubules were separated. The dispersed cells were centrifuged at 500 g for 5 min and then washed with phosphate-buffered saline (PBS). The second step of enzymatic digestion was carried out using the same procedure and enzymes (15 min). The isolated cells were washed again with PBS and filtered through a mesh with a pore size of 40 μm.

### Seminiferous tubule collection

The ice-cold testes were cut into four pieces and transferred to dishes containing full culture media. Under a stereomicroscope, the seminiferous tubules were then exposed by cutting into the connective tissue separating two adjacent tubules and pulling the connective tissue gently away. This mechanical isolation was done with fine forceps and then the suspension of seminiferous tubules was washed with PBS. The dissociated seminiferous tubules were suspended in α-minimum essential medium (αMEM; Gibco) including 10% knockout serum replacement (KSR) (Invitrogen), and then stored at 4 °C until use.

### Agarose gel preparation

**Fig. 1:**
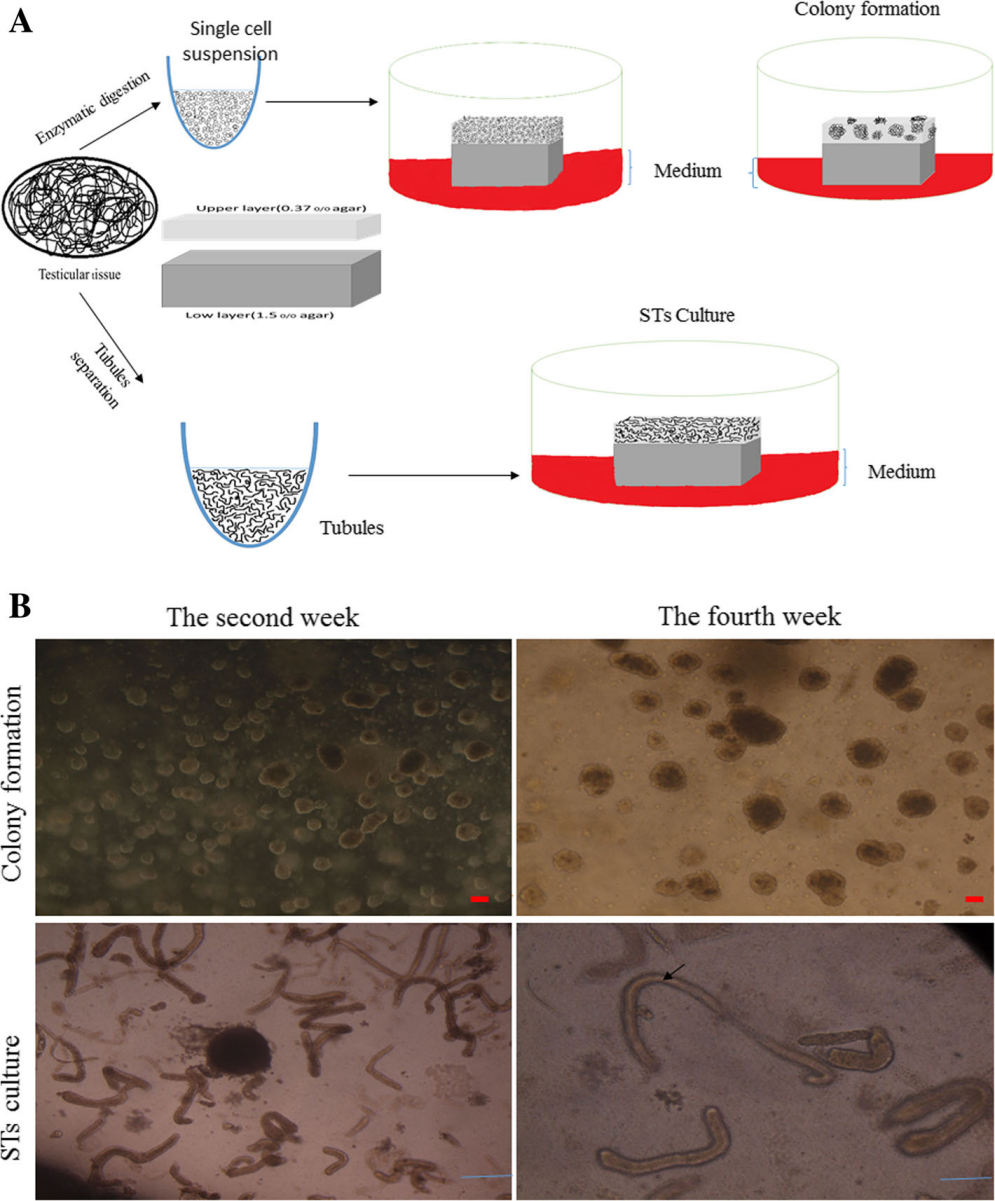
In-vitro spermatogenesis using culture of seminiferous tubules (STs) or testicular cells from 3- or 6-day-old mice. **a** Schematic presentation of experimental procedures. **b** Stereomicroscopic appearance of colony formation and seminiferous tubules. Arrow indicates complete canalization of seminiferous tubules in the fourth week. Scale bars in STs = 1 mm and in colonies = 50 μm

Our culture system was derived from SACS by modification and is composed of two layers of agarose: the soft layer (upper) with a concentration of 0.35% agarose, and solid layer (lower) with 1.5% agarose. The single cell or seminiferous tubule suspension was separately added to the soft/upper layer (0.35% w/v agarose) established on the solid/low layer (1.5% w/v agarose). This culture technique is a combination of the procedures used for organ culture and SACS [7, 8]. For this purpose, as shown in Fig. 1a, we dissolved 1.5 g of agarose (Fermentas, low melting point, #R0801) in 100 ml of distilled water and sterilized by autoclaving (2 atm, 121 °C, 20 min); 0.7% agarose solution was made in the same way. For the preparation of the base layer, each well of a six-well plate received 3 ml of 1.5% agarose solution and the plate was left for about 2 h at room temperature until the agarose gels became solid. After stiffening the gel, each well of a six-well plate had culture medium added (αMEM + 10% KSR) to soak the gel completely. To replace water in the gel with the medium, the plate was placed in an incubator overnight. For establishing the soft layer/upper, the pelleted testicular cells/STs were suspended in 0.5 ml 2× αMEM containing 2× 15% KSR, recombinant follicle-stimulating hormone (rFSH; 5 IU/l; Sigma), and 10^–7^ M testosterone (Sigma) and then mixed with 0.5 ml 0.7% agarose solution at 38 °C for preventing heat shock. After removing the old medium by aspiration, 1 ml of this mixture was placed on the base layer (1.5% agarose) in each well of a six-well plate and left for about 15 min at 4 °C until agarose gels of the soft layer became solid. Then, the entire two-phase gel in each well of a six-well plate was cut into pieces about 5 × 5 mm in size with a scalpel blade or spatula. Three parts were placed into each well of a six-well plate, adding new culture medium (α-MEM + 15% KSR, rFSH 5 IU/l, and 10^–7^ M testosterone) into each well to increase the height of the agarose gel to half or four-fifths of the pieces of the lower layer. All culture experiments were maintained in standard cell culture incubators at 34 °C and 5% CO_2_.

### Immunohistochemistry and immunocytochemistry for characterization of seminiferous tubules and colonies

For the characterization of spermatogonia (PLZF*,* INTEGRIN α6), spermatocytes (SCP3), spermatids (ACROSIN (ACR), and the Sertoli cell marker (VIMENTIN), the profile of protein expression was determined using immunocytochemistry (ICC) and immunohistochemistry (IHC) of colonies and frozen longitudinal section of STs, respectively (all antibodies were purchased from Abcam). After fixation with 4% paraformaldehyde for 24 h, the colonies and cryosections of STs were permeabilized with 0.4% Triton X100 (Sigma) and then blocked in 10% goat serum (Sigma). The colonies and sections were then incubated with primary antibodies for 2 h at 37 °C followed by washing in PBS. Subsequently, the secondary antibody labeled with FITC, diluted at 1:200 (Sigma), was added and incubated for 3 h. Control cells were treated under similar conditions except for the removal of the primary antibodies. Nuclei were stained with propidium iodide (PI; Sigma, Germany).

### Real-time polymerase chain reaction (PCR)

After 40 days of culture, the colonies and STs were removed from the agar using a denudation pipette and transferred into drops containing PBS at 39 °C in a CO_2_ incubator for 10 min. To remove the agar from colonies and STs completely, they were washed several times with PBS at 39 °C and then used for RNA extraction. The relative expression levels of *Plzf*, *Mvh* (mouse vasa homolog), *Integrin α6* (*Itga6/CD49f*) (undifferentiated genes), and *Scp3* (differentiated gene), were measured by real-time PCR. Total RNA was extracted by Trizol reagent (Ready Mini Kit, Qiagen, USA) according to the manufacturer’s instructions. Total RNA (1 μg) was applied for cDNA synthesis using a cDNA synthesis kit (Transcript First Strand cDNA Synt, Roche, USA) according to the manufacturer’s guidelines. Real-time PCR was carried out in 40 reaction amplification cycles, and Applied Bioscience 7500 fast with SYBR Green detection was used for the analysis. Melt curve analysis was performed after each run to detect the presence of nonspecific PCR products and primer dimers. All samples were normalized against glyceraldehyde-3-phosphate dehydrogenase (GAPDH) as an internal control, and the relative quantification of gene expression was determined using the comparative CT method (ΔΔCT).

### Statistical analysis

Statistical significance between the two data populations (single cell culture and seminiferous tubule culture) was evaluated using an unpaired, two-tailed Student’s *t* test in SPSS. Differences were considered statistically significant at *p <* 0.05.

## Results

### Colony development in the upper layer of the culture system

Isolated testicular cells were cultured in the upper layer. Colonies or aggregations appeared in the upper layer and showed a different growth pattern over time which was classified according to their size. Colonies of different sizes appeared after the second week. As shown in Fig. 1b, in the second week, the number of small (50–100 mm diameter) and medium (100–200 mm diameter) colonies was higher than the number of large colonies (diameter more than 200 mm). Conversely, the number of large colonies in the fourth week was higher. The solid layer (low layer) prevented the adhesion of cells to the bottom of the plate and resulted in maintenance of Sertoli cells in the upper layer and their connection to germ cells. In this way, the formation of colonies containing both Sertoli cells and germ cells in the upper layer was prompted by the lower layer.

### Morphologic assessment of seminiferous tubules

Seminiferous tubules embedded in 0.35% agarose were placed on the flat surface of agarose gel half-soaked in the medium. In the early days of culture, seminiferous tubules were visualized as solid and uncanalized cords with a narrow lumen which had a dark appearance. A significant increase in the diameter of tubules and steady canalization of seminiferous tubules over time indicated rapid growth of the tubule epithelium and the existence of secretion of Sertoli cells into tubules. We also observed that seminiferous tubule epithelium gradually turned clear, especially in the basement of the epithelium (see Fig. 1b).

### Spermatogenesis in colonies and seminiferous tubules

**Fig. 2:**
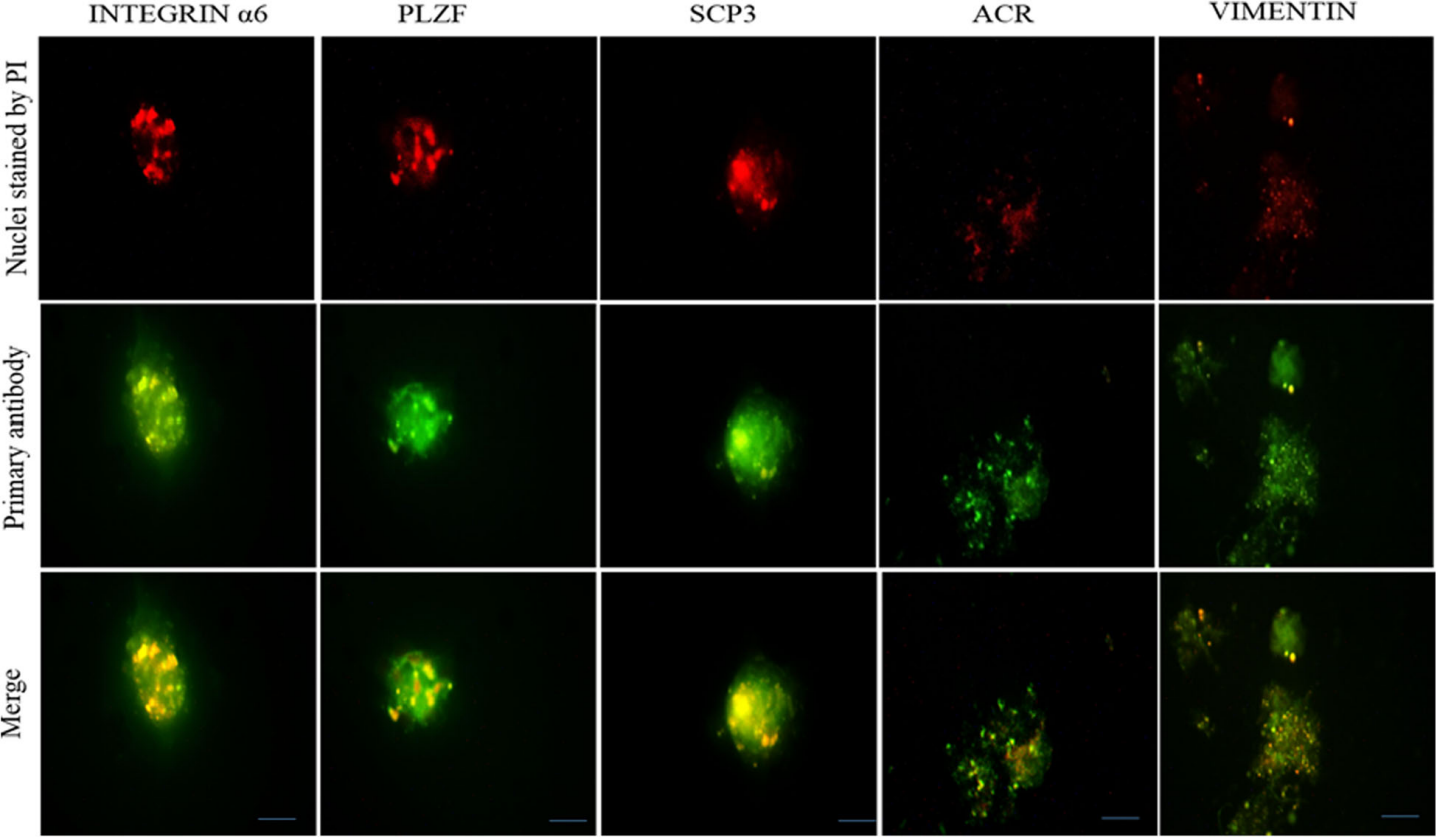
Immunofluorescence staining of colonies following 40 days of culture. Immunocytochemistry of colonies was performed by specific antibodies for different markers of germ cell development by immunofluorescence, including INTEGRIN α6, PLZF, ACROSIN (ACR), SCP3, and VIMENTIN. Scale bars =150 μm. PI propidium iodide

**Fig. 3:**
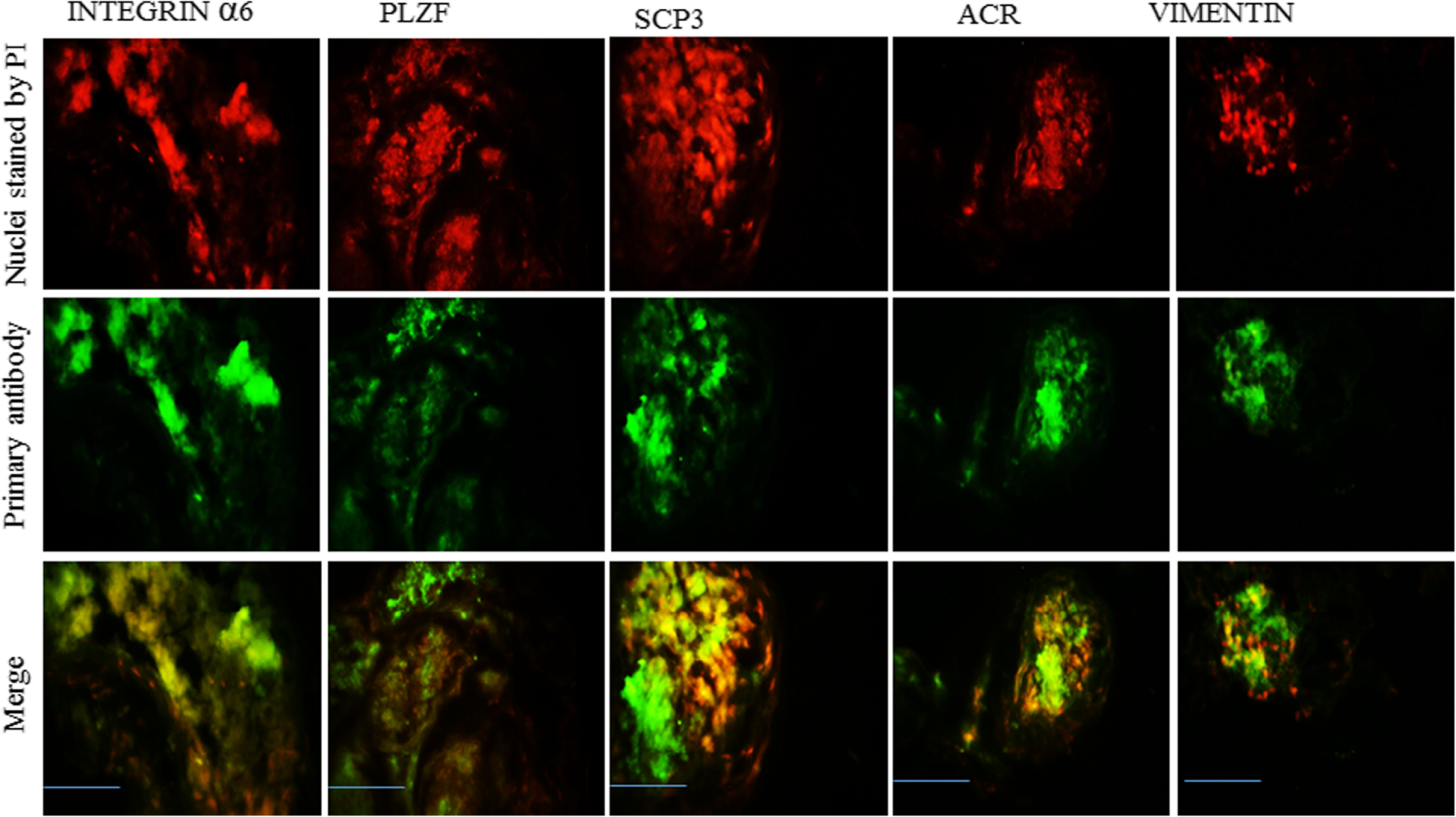
Immunofluorescence staining of frozen longitudinal sections of seminiferous tubules following 40 days of culture. Immunohistochemistry staining of frozen STs sections was performed by specific antibodies for different markers of germ cell development by immunofluorescence, including INTEGRIN α6, PLZF, ACROSIN (ACR), SCP3, and VIMENTIN. Scale bars =150 μm. PI propidium iodide

**Fig. 4:**
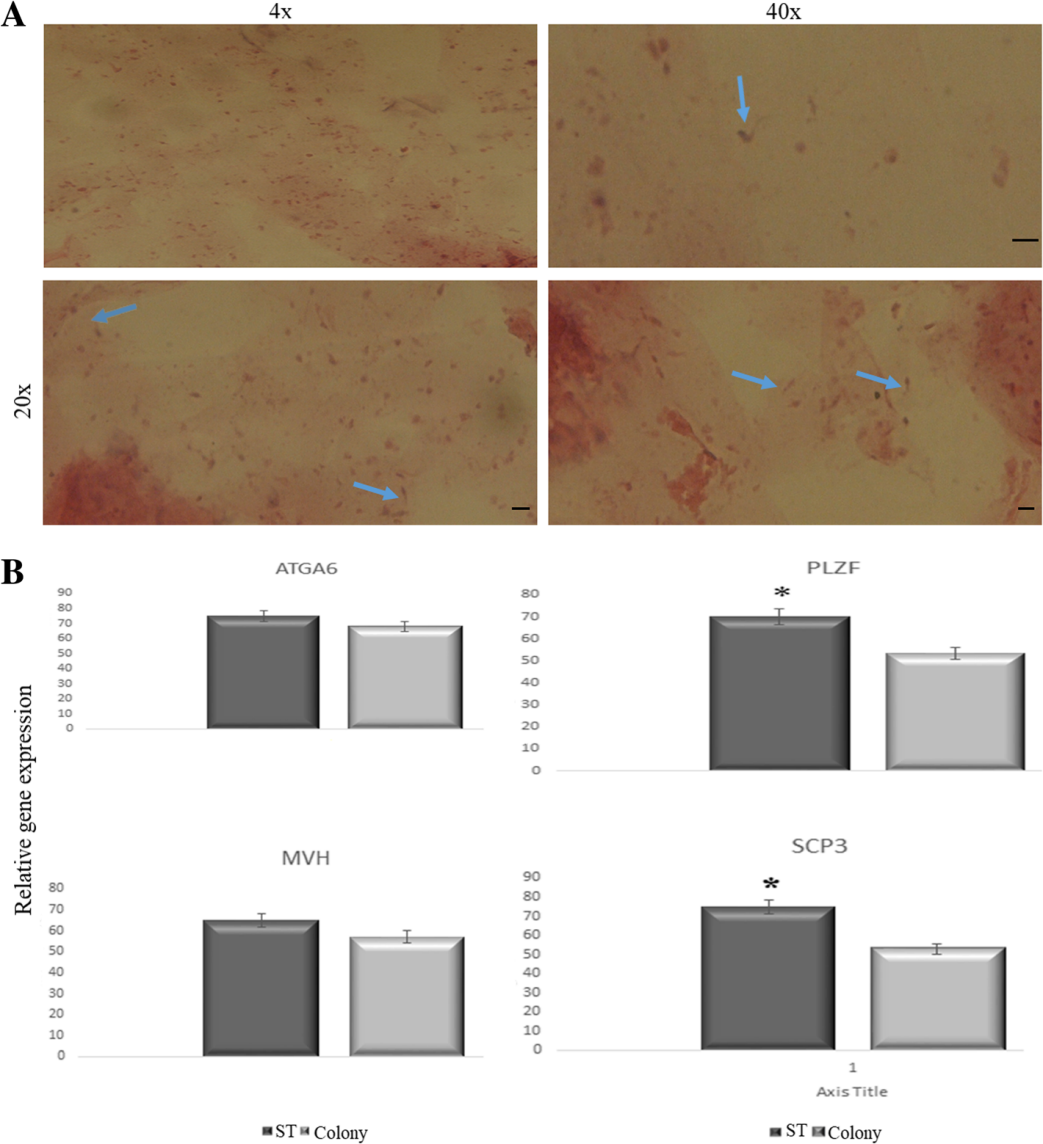
**a** Histological appearance of frozen longitudinal section of seminiferous tubules after 40 days of culture at different magnifications. Arrows indicate morphologically mature spermatozoa. Scale bars = 100 μm. **b** Comparison of expression of spermatogenesis genes in colonies and seminiferous tubules (STs) by real-time PCR. Data are expressed as means ± SD. *p < 0.05

For the identification of the spermatogenesis process in colonies and seminiferous tubules in the upper layer of the culture system, the expression levels of PLZF, INTEGRIN α6, SCP3, and ACR by ICC (used for colonies; Fig. 2) or IHC (used for STs; Fig. 3) were detected at the end of the culture period. In addition, to confirm the timing of entry of meiosis, the expression level of SCP3 was detected on the day 9 and at the end of the second week of cultivation in the STs and colonies, respectively, and meiotic progression was observed, corresponding well with the assumption that spermatocytes start meiosis at around 7.5 days postpartum (data not shown). PLZF and INTEGRIN α6 antibodies have been used in many studies as markers for pre-meiotic SSCs, and SCP3 and ACR are related to meiotic and the end-stage of meiosis onwards, respectively. These data demonstrated that spermatogenesis process had occurred. Furthermore, by performing real-time PCR to evaluate the genes related to proliferation and differentiation in colonies and seminiferous tubules, the results were confirmed. The results indicate that levels of *Plzf*, *Integrin α6* (*Itga6/CD49f*), *Scp3*, and *Mvh* expression in the seminiferous tubules group were higher than in the colonies group. However, the difference was significant only for *Scp3* and *Plzf* (Fig. 4b). The confirmed presence of morphologic spermatozoa using histological analysis of frozen longitudinal section of seminiferous tubules followed 40 days of culture indicated that the entire spermatogenesis process had occurred completely (Fig. 4a).

## Discussion

Although recent advances in the production of morphologically normal spermatozoa have created an expectancy of the clinical application of organ culture and SACS, limitations of these techniques should be discussed and considered further. In this study, with a special emphasis on the disadvantages of these methods, we developed a new technique that has somewhat improved the technical aspects of both methods. In fact, the features of both methods (organ culture and SACS) can complement each other. Since the medium is added to the upper layer of SACS in a 24-well plate, it causes a disturbance in gas diffusion and paracrine factor gradients around the colonies [15, 27]. Thus, the condition of the organ culture method, gas-liquid interphase, and gentle flow of medium through a half-soaked gel, can improve SACS with maintenance of the concentration of local factors and inhibition of hypoperfusion, especially for larger colonies. On the other hand, efficiency of the organ culture completely relies on the size and thickness of tissues [17, 28, 29]. To prevent hypoxic effects, very small testicular fragments have been used to facilitate the appropriate transport of gas and nutrients into the tissue. It seems that the culture of the mechanically isolated seminiferous tubules with only one epithelial barrier (58 μm in mice) can be valuable, especially in terms of ensuring appropriate perfusion and maintaining the three compartments of the seminiferous epithelium [17]. Our culture system using seminiferous tubules embedded in agar placed on a half-soaked gel can prevent many of the problems associated with culture of STs in two-dimensional culture systems, such as collapse of the tubule lumen, disruption of the epithelial structure, and the appearance of vacuoles [30–32]. The presence of spermatozoa in our culture indicates that the microenvironment necessary for the achievement of in-vitro spermatogenesis was well maintained and further indicates that this method can retain the special arrangements of the STs close to that in vivo [6]. Our findings are in line with results of a previous study in which spermatozoa were observed from culture of rat and human STs embedded in a hydrogel bioreactor [17]. On the other hand, this method can provide the appropriate conditions for aggregation and interconnection between Sertoli and germ cells. We have recently reported the positive effects of melatonin on the colonization of neonate mouse and spermatogonial stem cells in a SACS, along with leukemia inhibitory factor (LIF) and glia cell line-derived neurotrophic factor (GDNF). Supplementation of melatonin and these factors in the basic culture medium significantly increased SSC proliferation [33]. Considering the fact that aggregations of Sertoli and germ cells are the initial steps for the microenvironment and even tubular formation [23, 29], Elhija et al. showed that colonies that formed in the upper layer of SACS were free of somatic cells which were seen in the bottom of the plate [9]. Nonetheless, in our culture, the migration of Sertoli cells into the lower layer of SACS with more viscose agarose (1.5% vs 0.5%) was prevented so that Sertoli cells accumulated in the upper layer. The ICC results confirmed presence of vimentin (a Sertoli cell marker) in aggregations (Fig. 2). Because of the nature of the two phases of the culture system, it is possible that supplement factors or supporter cell lines can be added (Sertoli or Vero cells) to the soft agar phase, or even the bottom of the plate, to help differentiation into more developed spermatogenic stages [34]. This method may be useful for aggregations of testicular cells and the culture of STs. However, schemes should be considered to promote efficiency by adjusting the composition of the culture medium or material properties of hydrogels, such as conjugation with synthetic bioactive molecules like the Arg-Gly-Asp sequence (RGD peptides) [35].

## Conclusions

We conclude that our technique can provide a suitable microenvironment for interaction and aggregation of germ cells and Sertoli cells and also for culture of seminiferous tubules. Increased oxygen availability to larger colonies by an air-liquid interphase method and to seminiferous tubules by increasing the contact surface with air is shown to support in-vitro spermatogenesis into the later stages, although some aspects of the methodology need to be improved and the toxicity of air should be investigated.

## Abbreviations

Itga6: Integrin alpha 6
KSR: Knockout serum replacement
Mvh: Mouse vasa homolog
Plzf: Promyelocytic leukemia zinc finger protein
SACS: Soft agar culture system
Scp3: Synaptonemal complex protein
SSC: Spermatogonial stem cell
ST: Seminiferous tubule
